# Polygenic risk scores for objective and subjective social isolation and moderating effects of sex and material deprivation

**DOI:** 10.1192/j.eurpsy.2026.10473

**Published:** 2026-07-31

**Authors:** P. Cristóbal-Narváez, G. R. Alacid, B. Ramos-Josemaria, J. M. Haro, I. Gete-Alejandro, J. L. Ayuso-Mateos, M. Miret, J. Domènech-Abella, M. Fatjó-Vilas

**Affiliations:** 1 Institut de Recerca Sant Joan de Déu, Esplugues de Llobregat; 2 Parc Sanitari Sant Joan De Déu, Sant Boi de Llobregat; 3 Universidad Autónoma de Madrid, Madrid; 4 Universitat de Barcelona, Barcelona, Spain

## Abstract

**Introduction:**

Social isolation is shaped by genetic, psychological, and social factors. In the social domain, women and individuals in disadvantaged socioeconomic circumstances are at higher risk of both subjective (i.e. loneliness) and objective (i.e. social support) social isolation. In the genetic domain, previous research has shown that polygenic scores for loneliness and depression significantly predict subjective experiences of social isolation.

**Objectives:**

To investigate whether (1) polygenic scores for depression and loneliness are associated with social support and loneliness, and (2) whether these associations are moderated by social factors such as sex or material deprivation.

**Methods:**

We used data from the Edad con Salud 2019 cohort, a population-based study of non-institutionalized adults aged 18 and older residing in the provinces of Barcelona and Madrid. At baseline, data were collected from 3,002 participants between 2019 and 2021. In 2023, a follow-up survey was conducted through structured, computer-assisted personal interviews (CAPI). Biological samples (saliva) were collected to compute polygenic risk scores (PRS). The present study includes 618 participants with complete genetic and psychosocial data. Loneliness was assessed using the three-item UCLA Loneliness Scale, and social support using the three-item Oslo Social Support Scale. Material deprivation was measured using items developed by the Spanish National Statistics Institute (INE). Depression was measured using a structured diagnostic interview based on the Composite International Diagnostic Interview (CIDI). We examined the associations between PRS for depression and loneliness and the outcomes of loneliness and social support. We also tested whether these associations were moderated by sex or material deprivation using ordered logistic regression models.

**Results:**

In adjusted models, higher loneliness polygenic scores showed a significant association with poor social support. Depression polygenic scores did not show a significant main effect on social support, but there was a significant interaction with sex: among women, the probability of poor social support increased from 6.0% in the lowest depression PRS quartile to 18.9% in the highest quartile. For loneliness, there was a significant interaction between loneliness polygenic scores and material deprivation: among materially deprived individuals, the probability of reporting absence of loneliness symptoms decreased from 92.5.4% in the lowest loneliness PRS quartile to 60.2% in the highest quartile.

**Conclusions:**

Our results show that people with a genetic tendency toward loneliness not only feel lonelier but also receive less social support. This link is stronger in women with genetic risk for depression and in people living with material hardship, suggesting the importance of addressing both genetic vulnerability and social disadvantage.

**Disclosure of Interest:**

None Declared

